# Third-Generation Sequencing: The Spearhead towards the Radical Transformation of Modern Genomics

**DOI:** 10.3390/life12010030

**Published:** 2021-12-26

**Authors:** Konstantina Athanasopoulou, Michaela A. Boti, Panagiotis G. Adamopoulos, Paraskevi C. Skourou, Andreas Scorilas

**Affiliations:** Department of Biochemistry and Molecular Biology, Faculty of Biology, National and Kapodistrian University of Athens, 15701 Athens, Greece; konnath@biol.uoa.gr (K.A.); miboti@biol.uoa.gr (M.A.B.); Pskourou@biol.uoa.gr (P.C.S.); ascorilas@biol.uoa.gr (A.S.)

**Keywords:** long-read sequencing, PacBio sequencing, nanopore sequencing, single-molecule real-time sequencing, targeted DNA sequencing, direct RNA sequencing, metagenomics, epigenomics, epitranscriptomics

## Abstract

Although next-generation sequencing (NGS) technology revolutionized sequencing, offering a tremendous sequencing capacity with groundbreaking depth and accuracy, it continues to demonstrate serious limitations. In the early 2010s, the introduction of a novel set of sequencing methodologies, presented by two platforms, Pacific Biosciences (PacBio) and Oxford Nanopore Sequencing (ONT), gave birth to third-generation sequencing (TGS). The innovative long-read technologies turn genome sequencing into an ease-of-handle procedure by greatly reducing the average time of library construction workflows and simplifying the process of de novo genome assembly due to the generation of long reads. Long sequencing reads produced by both TGS methodologies have already facilitated the decipherment of transcriptional profiling since they enable the identification of full-length transcripts without the need for assembly or the use of sophisticated bioinformatics tools. Long-read technologies have also provided new insights into the field of epitranscriptomics, by allowing the direct detection of RNA modifications on native RNA molecules. This review highlights the advantageous features of the newly introduced TGS technologies, discusses their limitations and provides an in-depth comparison regarding their scientific background and available protocols as well as their potential utility in research and clinical applications.

## 1. Introduction

**Figure 1:**
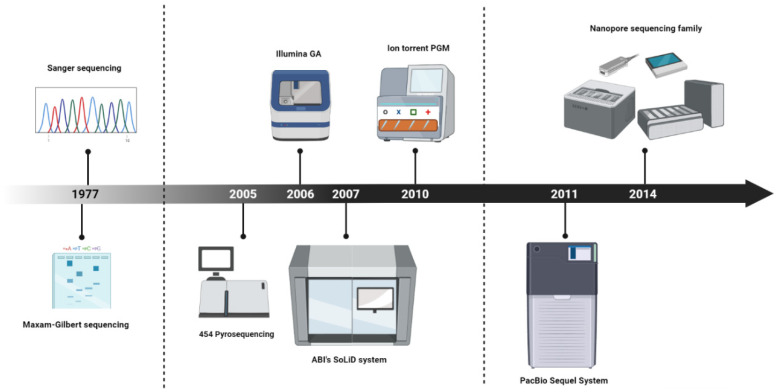
The milestones of sequencing development throughout the history of molecular biology.

Looking back in the late 1970s, when Frederic Sanger and his colleagues developed the first established method for DNA sequencing in the history of molecular biology [1,2], no one could imagine what would follow (Figure 1). Within a short period of time, before the Sanger sequencing method was released, Allan Maxam and Walter Gilbert had published their own work, presenting a novel DNA sequencing method [3], the first ever to be reported into the history of science. Being more complex and less scalable in comparison to the Sanger sequencing method, the “chemical sequencing method”, as dubbed by Maxam and Gilbert, did not have the anticipated impact on the scientific community, resulting in the ascendance of Sanger’s approach in the new era that had just begun. Sanger and Maxam–Gilbert sequencing represent the first-generation sequencing technologies and were the most common methods for DNA sequencing for almost three decades. These methods enabled the decoding of large genomes, with the striking point being the implementation of the Human Genome Project [4,5]. Although the first-generation sequencing technologies blazed a trail for deciphering genetic information, the low throughput and high cost of these time-consuming approaches gave rise to the need for the development of novel methodologies that would transform sequencing into a low-cost procedure characterized by high-throughput outputs.

The revolution of massive parallel sequencing started in 2005 with the presentation of the Roche 454 pyrosequencing system, introducing next-generation sequencing (NGS) technologies to the scientific community. This alternative approach of pyrosequencing enabled the implementation of DNA sequencing in a highly parallel manner, generating the first high-throughput technology [6]. Roche’s system, which was able to produce approximately 200,000 reads of 110 base-pairs (bp) [6], was then followed by the sequencing-by-synthesis-based Genome Analyzer platform from Illumina/Solexa in 2006 and ABI’s SOLiD system in 2007 [7,8]. The Illumina and SOLiD sequencers generated a greater number of reads (30 and 100 million reads, respectively), but the produced reads were only 35 bp long. In 2010, Ion Torrent released the Personal Genome Machine (PGM), a system that employs semiconductor technology and resembles Roche’s 454. The first PGM was able to generate approximately 270,000 reads, with an average length of 100 nt, while its working principle resulted in higher speed, lower cost, and reduced instrument size. The development of NGS technologies offered the opportunity for conducting thousands to many millions of sequencing reactions per run and marked the beginning of a new era in genome decoding. Due to their ability to produce a tremendous amount of data at an unprecedented speed and at low cost, NGS technologies have become a standard tool for many applications in basic and advanced research, as well as in clinical practice.

Shortly after the introduction of NGS, third-generation sequencing technologies (TGS) emerged. The two fundamental and distinctive features of the newly introduced TGS approaches, referred to as single-molecule sequencing (SMS) and sequencing in real-time, enable the sequencing of nucleotide molecules (DNA or RNA) without the need for PCR amplification of the template. They also allow the real-time analysis of the produced data. The ability of TGS to sequence DNA or RNA without any prior amplification of the template constitutes a major breakthrough, mainly because it ameliorates the biases introduced by PCR during the library construction. The first attempt at sequencing single molecules was made in 2009 when Helicos Bioscience launched HeliScope, a genetic analysis system performing single-molecule sequencing exploiting the phenomenon of fluorescence [9], and involved a library preparation workflow that was carried out prior to sequencing. Briefly, this methodology includes the shearing of the template into short DNA fragments (100–200 nt), which is followed by the polyadenylation of these fragments and the generation of poly(A) tails. Afterwards, the tailed DNA is hybridized to oligo-d(T) primers, which are immobilized on a disposable glass flow cell that includes 25 channels for sequencing. Prior to sequencing, treatment of the 3′ ends with terminal transferase enzymes is used for blocking the extension of the DNA. Finally, the identification of the DNA sequence is based on the emission of the light that is detected via imaging after the incorporation of a fluorescent nucleotide [10]. Although this method simplified the library preparation process, due to the avoidance of PCR amplification steps, it demonstrated serious limitations regarding the time-consuming sequencing, its high cost, the high error rates and, last but not least, the production of short reads (~32 bp).

In early 2011, Pacific Biosciences (PacBio) released their PacBio RS sequencer, giving rise to the first established single-molecule real-time (SMRT) sequencing technology [11]. Although the initial sequencer led to the generation of relatively short average read lengths (~1.5 kb) that were characterized by high error rates (~13%) [12], the technology was improved over the years, leading to the release of a new sequencer, the Sequel System, which was quickly established as the basic sequencing machine of PacBio for genome analysis. Over time, PacBio’s Sequel System obtained further improvements, resulting in the generation of two additional Sequel platforms, the Sequel II System and Sequel IIe System, with the latter being the most powerful platform among the members of the Sequel family [13].

In addition to PacBio’s methodology, TGS technologies were enriched by the development of an additional approach that is based on a totally different method compared to Sequel technology. More precisely, in 2014, Oxford Nanopore Technologies (ONT) introduced nanopore sequencing [14], which is based on the idea of using nanopores in a membrane to sequence single-stranded DNA or RNA molecules that was first proposed at the end of the 1980s [15]. Through the rapid evolution of ONT chemistries, a significant increase in throughput has been achieved, resulting in the establishment of Nanopore sequencing in multiple research fields.

## 2. Overcoming the limitations of NGS

Although NGS technology brought the revolution in sequencing, offering a tremendous sequencing capacity with groundbreaking depth and accuracy, it still demonstrates serious limitations, with the major issue being the generation of short reads. The so-called “short-read sequencing” that characterizes all NGS platforms requires the usage of specialized bioinformatics tools and complicated post-processing pipelines, making the manipulation of the high-throughput data harder and increasing the average time of analysis. NGS technologies’ limitations were overcome with the introduction of TGS, a novel methodology that marked the beginning of a new era in sequencing. Distinct from NGS platforms that generate relatively short reads (up to ~600 nt), TGS is characterized by improved sequencing chemistry, leading to the production of long reads, with an average length of more than 10 kb [16,17]. The significant increase of the generated sequencing reads’ length is considered the most advantageous feature of TGS technologies since it drastically improved the quality of genome assembly and the analysis of genome structures [18,19]. More precisely, longer read lengths act as more representative elements of chromosomes and, consequently, produce more contiguous reconstructions of the genome [18]. According to variation analysis studies, long reads paved the way for an easier characterization of large insertions, deletions, translocations and other structural changes that may exist throughout the genomes.

The distinguishing features of TGS technologies are, single-molecule sequencing and real-time sequencing. As opposed to NGS, where PCR amplification is an essential step of the experimental procedure, the absence of PCR amplification characterizing TGS approaches allows less bias and more homogenous genome coverage [17]. Except for genomic DNA sequencing, long-read technologies are ideal for studying transcriptomes, resulting in the identification of novel full-length transcript variants and gene fusions that could not be detected using NGS approaches [19]. In addition, TGS platforms have been established as the “gold-standard” technology for whole-transcriptome sequencing since they enable an easier and more efficient exploring of complex transcriptomes without the need for specialized assembly bioinformatic tools.

Notably, short-read methods normally involve the usage of large equipment and laborious experimental procedures, as well as extensive bioinformatics analysis. These characteristics of NGS approaches make the experimental procedure more time-consuming and complicate the post-processing analysis [20,21]. In contrast, TGS platforms have developed new sequencing protocols and, in this way, simplified library construction processes, minimizing their preparation time and making sequencing runs an ease-of-handle and time-saving procedure. As for the required laboratory equipment, the revolution has developed with ONT, which changed our perception regarding the size of a sequencing machine, launching pocket-sized sequencers that enable the user to construct a library and perform a sequencing run on the field, right after the collection of an environmental sample [22].

## 3. The Revolution of PacBio Sequencing

**Figure 2:**
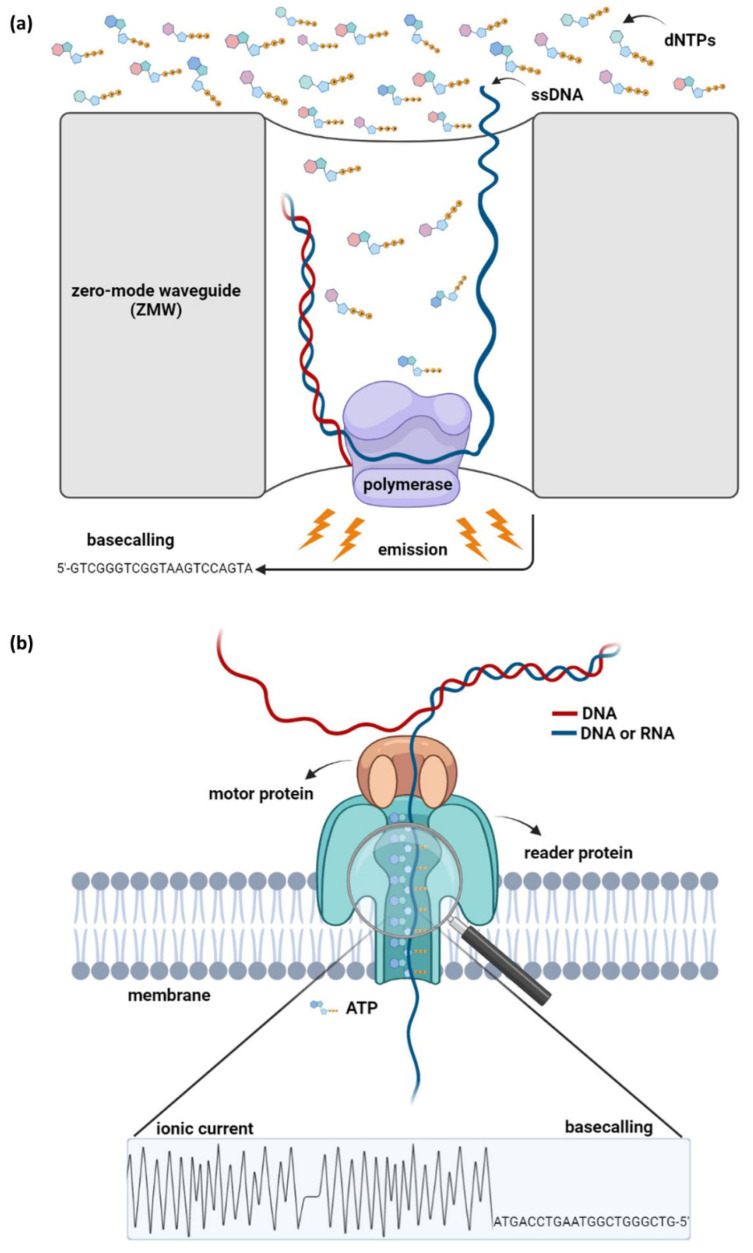
Scientific background of both third-generation sequencing technologies. (**a**) PacBio sequencing. The method is based on DNA sequencing by synthesis. The mobile single-stranded DNA is attached to the stable polymerase, which catalyzes the incorporation of dNTPs in a newly synthesized complementary DNA strand. The reaction occurs in specially designed zero-mode waveguides (ZMWs) that enable the observation of the emitted light. Afterwards, the signal is translated into nucleotide sequence, a procedure known as basecalling. (**b**) Nanopore sequencing. The method relies on the guidance of a single-stranded DNA or RNA molecule to a nanopore, a reader protein that detects alterations in the electrical current, which occurs during the passing through of the DNA/RNA.

The PacBio sequencing method, also referred to as single-molecule-real-time (SMRT) sequencing, is the first nanosensor-based technology introduced by Pacific Biosciences (PacBio) in the early 2010s [23,24]. PacBio technology exploits the properties of DNA synthesis and allows the identification of DNA molecules up to 50 kb or more [25]. Previous sequencing methodologies that are also based on DNA replication are characterized by the DNA-binding capacity of polymerase enzymes [26]. In NGS techniques, polymerase moves along the template DNA and provides sequencing by catalyzing the incorporation of dNTPs in a new complementary DNA strand. In contrast, the idea of SMRT sequencing is based on the immobilization of a DNA polymerase molecule in each well of a specially designed silicon chip (“SMRTcell”), while DNA is the mobile molecule. Synthesis reactions are measured within thousands of wells that contain microscopic chambers, called zero-mode waveguides (ZMWs) [26,27]. The ZMW sensors prevent the propagation of the light that is emitted by the incorporation of phosphate-labeled dNTPs at the elongated strand, whereas a system that includes both a laser and a camera detects and records the signal generated into the lower part of the sensors [27,28]. PacBio platforms allow continuous optical observation of the illuminating light and enable the simultaneous and parallel detection of thousands of single-molecule sequencing reactions. For the conduction of sequencing, a special, circular double-stranded DNA adapter, named “SMRTbell”, is necessary; thus sample preparation comprises the connection of “SMRTbell” to the DNA target. In each ZMW, the DNA polymerase can elongate complementary strands of large templates multiple times through the presence of “SMRTbell” by traversing the circular DNA in the 5′→3′ direction (Figure 2a) [27,28].

All PacBio platforms are based on single-molecule, real-time sequencing technology that detects alterations in light emission when a nucleotide is incorporated by the DNA polymerase. Over the years, many SMRT sequencers have been designed and made available by PacBio; however, the previous devices, such as PacBio RS II, are replaced by the Sequel System Family instruments that share common, optimized features for example updates on previous chemistry techniques, robotic workflow, monitoring runtimes, touchscreen, integrated software and controlling the capacity of every run. The Sequel System has the capacity to produce a total data output of up to 7.6 Gb, with reads of an average length that outreach 10,000 nt. The Sequel System was released in 2015 and is the first member of the Sequel Family [29]. This platform can reach up to 20 h of sequencing runs and has 1 million ZMWs that can generate up to 500,000 high accurate reads with an average length of 15 kb. On the other hand, the Sequel II System performs sequencing up to 30 h and features more than eight times the sequencing data output compared to Sequel System (Table 1). Additionally, it supports 8 million SMRTcells; thus it can provide up to 4,000,000 long reads with the benefit of greater accuracy at a significantly reduced project cost. The Sequel IIe System is the newest PacBio’s platform that performs sequencing in 8 million ZMWs and generates up to 4,000,000 reads in a run that can reach up to 30 h [13]. The innovative features of the Sequel instruments include advanced data processing, reduction of computational cost and fast data transfer, emerging these systems as the ascendant PacBio’s sequencers in contemporary laboratories.

## 4. The Concept behind Nanopore Sequencing

The pioneering technology of nanopore sequencing was first proposed in the late 1980s; however, the first nanopore-based sequencer became available in 2014 by Oxford Nanopore Technologies (ONT). Nanopore technology relies on detecting changes in the electric current, provoked by the disorder of nanopore proteins when DNA or RNA strands pass through them. In contrast to NGS methodologies that detect secondary signals, nanopore technology directly identifies the changes in the electric current that are produced in real-time during the sequencing run. The Nanopore system consists of nano-sensors, a special structure of “channels” through which DNA penetrates. Double-stranded DNA molecules are denatured, and, subsequently, a single strand enters the channel. Then, an auxiliary protein, called a motor protein, directs the single-stranded DNA, and facilitates its passage through the pore. This leads to the disturbance of the channel’s electric current, an alteration detected by a sensor protein known as a reader. The alterations of the ionic current are distinctive for each nucleotide, generating a unique signature for each base. The process of translating the electrical signal into nucleotide bases is called basecalling. However, ONT systems do not identify individual bases since the observable current is determined by short nucleotide sequences [30]. These sequences of approximately five bases, are called k-mers; thus, more than 1000 different signals, one for each micropolymer (k-mer), can be detected (Figure 2b).

ONT performs real-time data streaming from the first 2 min of sequencing and enables the production of ultra-long sequencing reads (>4 Mb) [31]. In contrast to the time-consuming processing steps of NGS, TGS has diminished the sluggish pre-sequencing library construction, allowing high-speed sample preparation protocols, with a minimum time of 10 min for library construction. The special features of the flow cells provided by the ONT sequencing platform have hastened sequencing approaches. Each flow cell accommodates 512 different channels (nanopores) that perform sequencing simultaneously, leading to the generation of a tremendous data output within a short period of time [32,33]. Although the available flow cells have R9 chemistry that enables >98.3% accuracy per single molecule, new R10 chemistry with single-molecule accuracy >99% is currently under development. A hallmark of ONT platforms is the newly introduced type of chemistry that can be used for assembling information from both strands of the template DNA. In detail, besides the “one-directional” (1D) sequencing approach where only one strand passes through the membrane and is being sequenced, in the 1D^2^ system, a primary adapter is used to promote the second strand through the pore after the passage of the first one and, thus, enabling the sequencing of both strands [17,34,35].

Moreover, nanopore sequencing devices demonstrate unequivocal features, which include the type of the starting material (DNA, cDNA or native RNA), the option for multiplex sequencing and the wide range of the initial amount per TGS library that ranges from 10 pg-1 mg. A mini-review of the exclusive characteristics of each platform is presented below.

MinION™ devices are the only available portable, pocket-sized sequencers that allow real-time and rapid sequencing that can reach up to 420 bases/s, leading to a maximal yield of 50 Gb per flow cell in a 72-h run (Table 1) [33,34,35]. Currently, there are two versions of MinION™ devices: MinION™ Mk1B and Mk1C, both compatible with MinION™ flow cells. Moreover, both platforms are compatible with the Flongle adapter, enabling rapid and low-cost real-time sequencing on lower capacity, single-use flow cells that can generate up to 1.8 Gb yield [36]. It should be mentioned that MinION™ Mk1C, the latest “all-in-one” sequencer, encompasses a specially designed MinKNOW software with straightforward hardware, powerful GPU-based computing capacity, network connectivity and a high-resolution screen, enabling sequencing in any environment [37]. The aforementioned features have established MinION™ as the most widely used ONT’s sequencer by conventional laboratories. Furthermore, GridION™ constitutes a compact benchtop system, which is designed to perform sequencing in one to five MinION™ flow cells or Flongle adapters, either individually or simultaneously, leading to the production of a maximum data output of 250 Gb, thus enhancing the design flexibility of the sequencing of multiple DNA/RNA samples (Table 1). Among the special features of ONT devices, GridION™ offers real-time processing of the sequencing data and analysis through an integrated computing system that includes the MinKNOW software and requires only network connectivity and a power source [38]. For a greater extent of massive parallel sequencing, ONT has designed PromethION™, a sequencer that allows up to 48 sequencing runs concurrently, permits real-time local basecalling and analysis and generates outputs up to 245 Gb per flow cell, depending on sample and library preparation method (Table 1). Three additional platforms are yet to be released by ONT: PromethION™P2, SmidgION™ and Plongle™. PromethION™P2 relies on PromethION™ design and aims to generate Tb of data, whereas SmidgION™ is expected to be the smallest sequencing device that can be connected to a smartphone. Plongle™ is designed for smaller experiments and has a 96-well plate format.

## 5. Bioinformatics Tools for Downstream Analysis

The massive amount of data generated by third-generation sequencing approaches encompasses multidimensional information for sequencing reads, including real-time kinetic characteristics [39]. Although both PacBio’s and ONT’s generated files include a great amount of data that increase their size to a large extent, the estimation of an average file size would be quite difficult. The wide range of the size is due to multiple criteria such as the sequencing device, the implemented workflow, running time, as well as the input amount and, thus, a suggested average size would not be representative.

Files used for data storage are based on the hierarchical data format 5 (HDF5) standard, since PacBio has adopted the h5 format, whereas the ONT platforms use the FAST5 file format [32,33]. HDF5 files contain the maximal of information generated by a sequencing run and differ from the FASTQ output that is provided by NGS approaches; thus the usage of new bioinformatics tools for the analysis of TGS data outputs is necessary. The first step for data analysis is the conversion of raw data into a nucleic acid sequence, a process known as basecalling. In PacBio’s h5 files, the translation of kinetic information into nucleotide sequences follows the circular consensus sequencing (ccs) workflow and produces consensus sequences of high accuracy (>99%), called HiFi reads [40]. ONT devices integrate algorithms that produce basecalled FAST5 or FASTQ files, such as Guppy software, which is currently the eminent basecaller used by ONT and performs both live and on-demand basecalling [30,33]. Updates on basecalling software have reduced the basecalling error rate in both PacBio and Nanopore sequencing platforms to <1% and <5%, respectively [41,42]. Additionally, the quality control step that is based on predefined metrics, categorizes the sequencing reads in high- and low-quality. Indicatively, LongQC is a reliable tool that is used for TGS datasets and evaluates the quality of the reads [43].

The derived high-quality reads are suitable for downstream analysis that involves a bioinformatic pipeline almost identical to the well-established pipelines for the NGS datasets. In detail, the adapter sequences are trimmed out from the initially created FASTQ files, and, subsequently, the generated trimmed FASTQ files are aligned to a reference genome. Then, the produced SAM files that include information from the aligned reads are converted into BAM or BED files for visualization by Genome Viewers, such as the Integrative Genomics Viewer (IGV) [44]. Additionally, Canu [45] and Prowler [46] represent filtering tools for trimming the adapter sequences whereas, Minimap2 [47], GraphMap [48] and BWA-MEM [49] are conventional aligners for TGS datasets, being eligible to map QC passed reads to a reference sequence.

The innovation of long sequencing reads, provided by TGS platforms, allows highly accurate de novo assembly [50,51]. Nonetheless, the middle step of reading error correction is rather necessary to diminish the potential biases from the downstream analysis steps. Long reads can be corrected by two different approaches. The first approach involves de novo error correction by the self-alignment of the overlapped reads, while the second one exploits short reads (Illumina’s) for correction of long reads [50]. Under that prism, Jabba [52], LoRMA [53], Nanocorr [54] and Nanocorrect [55] were designed to correct the long reads [50]. Moreover, most of the published long-read assemblers follow an overlap-layout-consensus (OLC) approach that involves raw read mapping, error correction, pre-assembly, consensus build-up and consensus polishing. Namely, Racon [56], Canu [45] and miniasm [57] constitute the most well-known tools used for TGS read assembly.

Additionally, handling the huge amount of data generated by TGS approaches demands multitools capable of manipulating and extracting the maximal information. Although several tools have accomplished a notable breakthrough in terms of bioinformatics analysis, further computational pipelines are required to enhance the analysis capacity of the obtained high-dimensional data.

## 6. Applications

The evolution of third-generation sequencing paved the way for the development of new research areas through the major sequencing methods of DNA-seq and RNA-seq [17]. The establishment of novel approaches that enabled the sequencing of non-amplified DNA or RNA templates steered the scientific community towards the research fields of epigenetics and epitranscriptomics.

### 6.1. DNA Sequencing

**Figure 3:**
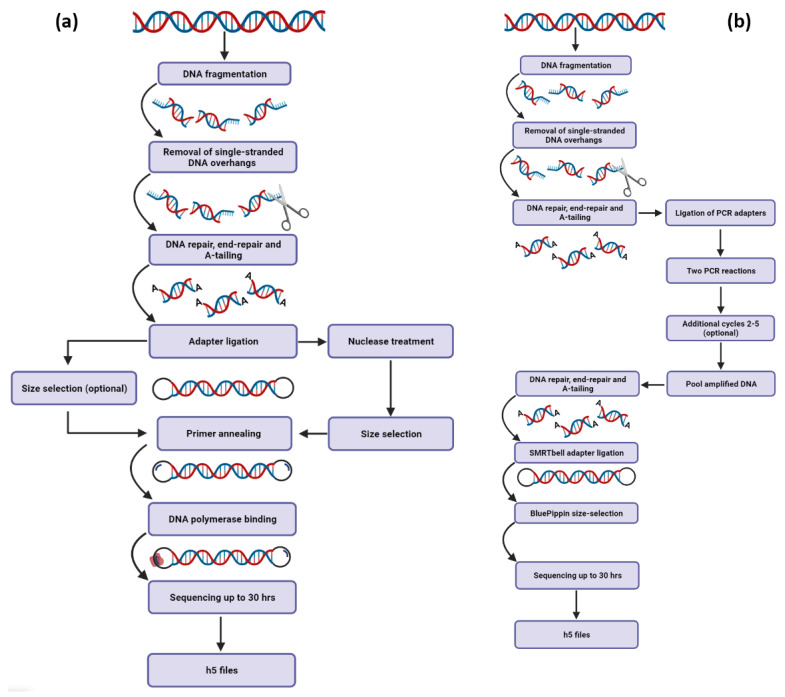
Schematic demonstration of PacBio’s main workflows for DNA sequencing. (**a**) The SMRTbell Express approach uses gDNA as a template for the construction of HiFi SMRTbell libraries that are ideal for variant detection and de novo assembly applications. When handling larger inserts, a permutation of the approach is suggested. An additional step that includes nuclease treatment is necessary for further DNA segmentation. (**b**) In cases of ultra-low DNA inputs, PCR reactions must be conducted to achieve higher quantity of the starting material.

**Figure 4:**
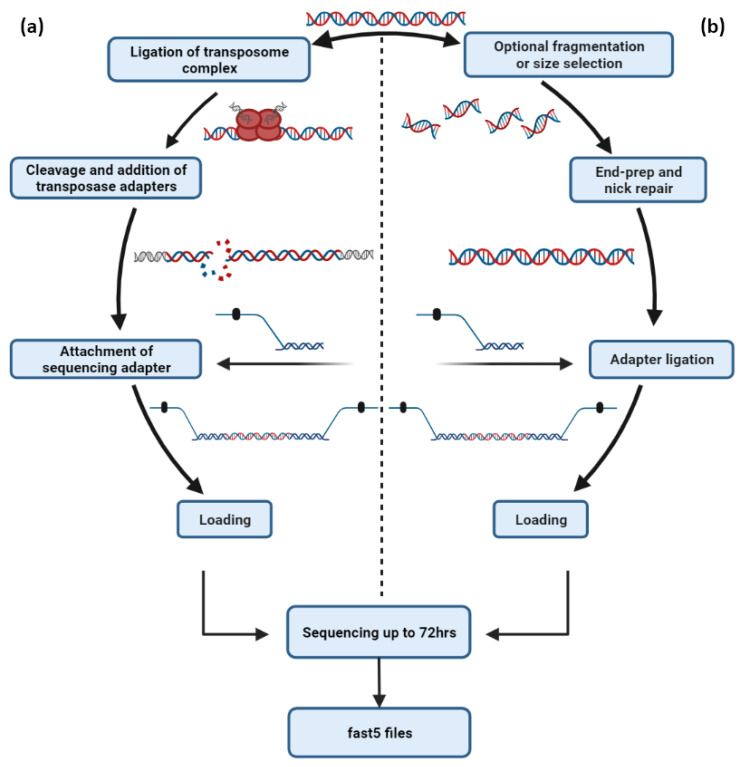
The most representative DNA-sequencing workflows of Oxford Nanopore Technologies. (**a**) For minimal library preparation time, ONT provides the Rapid Sequencing workflow, which exploits the innate qualities of transposase for the cleavage of genomic DNA and the subsequent adapter ligation. (**b**) For maximum throughput, ONT has developed the sequencing by ligation workflow, which includes DNA end repair and attachment of sequencing adapters for the sequencing of genomic DNA or specific amplicons.

Third-generation sequencing platforms have already simplified the sequencing of entire human, animal, plant and microbial genomes by producing long sequencing reads with greater overlaps, significantly facilitating the de novo genome assembly process [54,58]. Both PacBio’s (Figure 3) and ONT’s (Figure 4) primary workflows consist of DNA template preparation, purification and adapter ligation steps that lead to the employment of fast and real-time DNA sequencing (Table 2). Numerous studies have already revealed that these platforms possess the ability to decipher even the most challenging regions of complex eukaryotic genomes [42], reveal structural variants that were non-detectable by previous sequencing chemistries [59] and implement telomere-to-telomere assemblies of whole chromosomes [60]. TGS will soon transform the assembly of diploid genomes into a routine process, which will revolutionize genomics by revealing the atlas of human genetic variations, elucidating some of the missing heritability and leading to the identification of novel disease-related molecular mechanisms.

De novo assembly through whole-genome sequencing (WGS) approaches have introduced revolutionary changes in genomic research and has broadened the boundaries of DNA studies. In brief, DNA-seq application enables the correction of existing reference genomes and the characterization of previously undefined genomes. Moreover, sequencing of native DNA can be employed for mutational analysis, pathogen detection, identification of epigenetic modifications, determination of haplotypes and alleles, identification of a substantial number of variations and discrimination of complete repetitive regions from pseudogenes (Table 2) [29,61,62,63]. Thus, comprehensive genome profiling can provide insights into every area of life and biology, such as clinical tests (e.g., HLA typing) [29,64], drug discovery [65,66], identification of new disease-related genes [67], cancer research [68], pre-implantation genetic diagnosis and counseling [29].

#### 6.1.1. Whole-Exome Sequencing (WES)

In contrast to WGS, exome sequencing targets the protein-coding regions that represent only <2% of total genomic DNA. Consequently, WES is characterized by higher depth and coverage as compared to WGS, allowing an increased number of samples to be sequenced in a single run, on the point of lower cost. WES constitutes an approach with critical significance in multiple research fields, which can be applied both for research and clinical/diagnostic purposes, allowing the detection of functional disease-related variants, the identification of modified bases in coding sequence regions, as well as the detection of CNVs (Table 2) [69].

#### 6.1.2. Targeted Sequencing

**Figure 5:**
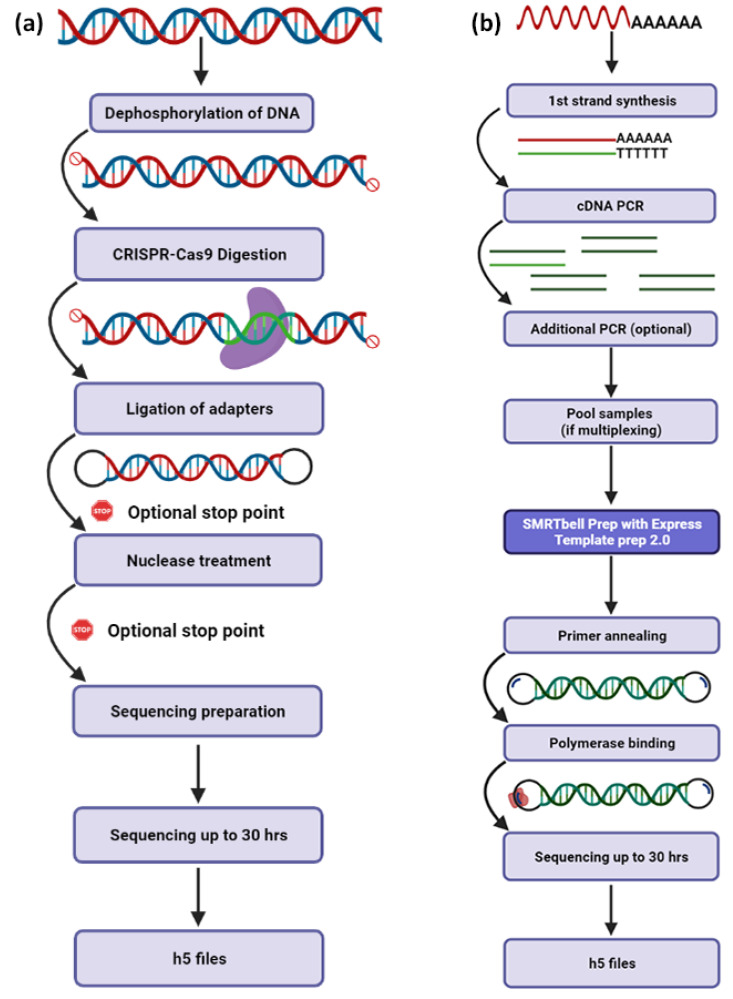
Schematic demonstration of fundamental workflows for PacBio’s libraries’ construction. (**a**) The process describes the sequencing of targeted regions of genomic DNA without the need of amplification. The innovation lies in the usage of the CRISPR-Cas9 system for template enrichment. (**b**) Iso-seq™ SMRTbell^®^ workflow is designed to enable the characterization of full-length transcripts from total RNA. The procedure involves first-strand cDNA synthesis and PCR amplification, followed by the classic workflow for DNA sequencing library construction.

The technologies of TGS platforms have revolutionized sequencing not only on the genome- and transcriptome-wide level but also in terms of targeted sequencing, facilitating the accurate identification of specific genomic or transcriptomic regions of interest. Under that prism, PacBio technology offers an efficient approach for sequencing targeted genomic regions that requires a total of 5000 ng non-amplified genomic DNA and at the same time exploits the CRISPR-Cas9 system (Figure 5a, Table 3). The first step involves the detection of a specific recognition site by Cas9, which is associated with two guide RNAs, and subsequently, the enzyme cleaves the recognized site, whereas the final construction step of SMTRbell templates is the ligation of the sequencing adapters (Figure 5a). It should be mentioned that this approach enables the simultaneous sequencing of multiple targets by using different guide RNA pairs, thus significantly reducing the number of sequencing runs as well as the overall sequencing cost. Notably, PacBio supports two additional approaches that involve PCR steps for amplicon sequencing.

Besides the CRISPR/Cas 9-based approach, ONT provides PCR and PCR-free approaches for targeted sequencing of genomic regions. Although the main advantage of ONT’s CRISPR/Cas 9 sequencing system is the wide spectrum of the input requirement (1–10 μg), multiplexing options are not available. The amplification-free approach involves an initial optional fragmentation step and is based on the ligation of sequencing adapters in a specific region of interest that has already been treated by end-repair and nick-repair enzymes (Figure 4b). The PCR-free ligation workflow requires 1000 ng dsDNA as starting material, is suitable for the detection of multiple target regions, allows the sequencing of barcoded samples in a single run and enables the highest throughput that reaches up to 50 Gb per flow cell. For PCR-based workflows, an initial amount of 100 ng dsDNA is amplified, supporting targeted sequencing with multiplexing options (Table 3).

Specifically, hard-to-amplify genomic regions, low complexity regions, such as repetitive regions and promoters, as well as variations such as rare SNPs, SNVs, haplotypes and structural variants (Table 2), can be detected in highly accurate long reads [70,71,72]. Real-time targeted sequencing is in full swing, aiming to contribute as a routine tool for microbiology and infectious diseases, biomedical research, plant and animal research, the major fields in which targeted sequencing can be applied [73,74].

#### 6.1.3. cDNA Sequencing

Third-generation sequencing has emerged as the “gold-standard” technology for cDNA sequencing. The long reads produced by TGS platforms have the capability to identify full-length mRNAs or even fusion transcripts without the need for assembly (Table 2). Notably, PacBio enables the indirect sequencing of RNA molecules by preparing and sequencing cDNA libraries using the innovative approach of Iso-Seq Express Template Preparation (Figure 5b). Briefly, this methodology requires 300 ng total RNA, which are used as a template for first-strand cDNA synthesis, followed by cDNA amplification steps. Afterwards, the amplified cDNA is used as input for the SMRTbell library construction workflow, and finally, cDNA sequencing is performed, allowing the identification of full-length transcripts up to 10 kb (Figure 5b).

On the other hand, nanopore cDNA sequencing is also a useful approach for transcriptome profiling and the identification of alternative splicing events. It should be mentioned that in cases of limited initial quantities of poly A+ RNA, ONT has developed an approach (PCR-cDNA sequencing) that requires just 1 ng of poly A+ RNA as starting material and generates sequencing reads that represent enriched full-length cDNA molecules (Table 3). This is achieved by reverse transcription of the initial poly A+ RNA and template-switching using a specific primer, cDNA amplification of the created cDNA molecules using primers targeting the attached synthetic oligonucleotides (oligo-dT adapter primer and template-switching primer), and finally, adapter ligation to the amplified cDNA ends. This is without a doubt one of the most fundamental advantages of TGS over NGS since both Illumina and Ion Torrent platforms require a substantial amount of RNA as starting material, which in cases of challenging samples (e.g., wastewater samples) may constitute the most serious limitation.

**Figure 6:**
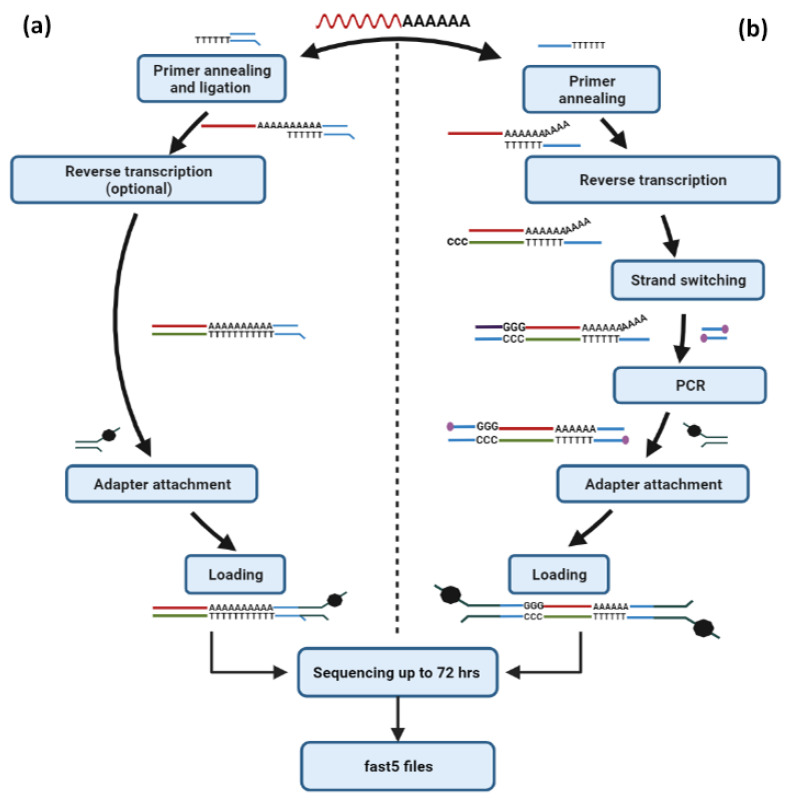
RNA-based sequencing protocols offered by Oxford Nanopore Technologies. (**a**) The Direct RNA sequencing approach enables the direct sequencing of RNA molecules without the need for amplification of the template and can be implemented with or without reverse transcription of the native RNA that is intended to be sequenced. (**b**) For high-throughput analysis of full-length transcripts, the PCR-cDNA sequencing approach is highly recommended, which requires poly A+ selected RNA as starting material and involves a reverse transcription step, cDNA amplification, adapter ligation and eventually sequencing.

Besides this approach, ONT’s direct cDNA sequencing method constitutes a reliable choice to perform the sequencing of cDNA molecules without amplification (PCR-free) but requires a higher input amount (100 ng poly A+ RNA). Direct cDNA sequencing workflow is based on reverse transcription of the poly A+ RNA, adapter ligation to the derived cDNAs and sequencing. Although this approach needs an increased amount of RNA as starting material and at the same time produces less data than the PCR-cDNA sequencing method, the avoidance of PCR leads to the generation of raw sequencing data that do not contain any PCR bias (Figure 6b). As a result, the direct cDNA sequencing approach is the ideal method for performing differential expression analysis of mRNAs in a sample of interest but will not have a high impact on the identification of novel mRNA transcripts that will be less abundant.

### 6.2. Nanopore-Based Direct RNA Sequencing

The establishment of ONT real-time direct RNA-sequencing approaches has efficiently dissected many aspects of human, plant and animal transcriptome complexity. This highly innovative approach directly sequences poly A+ RNA molecules that pass through the nanopores, and since it is unavailable through any NGS platform, it has emerged as an outstanding methodology for the study of mRNAs. The direct RNA sequencing method involves the ligation of an adapter targeting the poly-A tail of mRNAs and can be employed with or without a reverse transcription step and at the same time does not require any PCR amplification stage (Figure 6a). Notably, although the implementation of direct RNA sequencing without the reverse transcription step is less time-consuming and therefore enables a rapid sequencing workflow, it often demonstrates less throughput, and for this reason, the employment of the reverse transcription step is highly recommended. This is quite expected since the generation of RNA:cDNA hybrid molecules significantly improves the stability of the native RNA. In any case (native RNA molecules or RNA:cDNA hybrids), the adapter attached to the motor protein is specifically ligated to the RNA strand, and therefore only the RNA molecules are passing through the nanopores. Despite the innovative features of direct RNA, this approach requires a substantial initial amount of poly A+ RNA (500 ng), whereas it offers significantly less output as compared to both direct cDNA and PCR-cDNA methodologies.

Direct RNA sequencing is an efficient choice for the detection of full-length mRNAs and the identification of novel transcripts that can enlighten alternative splicing features and patterns [75,76,77]. Moreover, it allows accurate isoform quantification, characterization of polyadenylation sites, identification of promoters and splice sites, but most importantly, the detection of RNA modified bases (Table 2) [78,79,80,81]. This straightforward application has the potential to achieve revolutionary changes in personalized medicine, gene therapy, pharmacogenomics, viral and microbial medical sequencing, epigenetics and cancer research [76,82,83,84,85]. Thus it is expected to label the near-future of RNA research.

#### 6.2.1. mRNA Sequencing

The study of transcriptomes, which reflect the activity of genes in every cell, is vital for unraveling the relation between genomes and phenotypes. Besides the two well-known methods that were used for transcriptome analysis for years, the serial analysis of gene expression (SAGE) [86] and the DNA microarray [87], the development of RNA sequencing (RNA-seq) using NGS technologies (Mortazavi et al., 2008) rapidly became the ideal choice. Although RNA-seq through NGS demonstrated critical breakthroughs, including the increased depth and coverage at the transcriptome-wide level, it still required PCR amplification of the template, and eventually, it involved sequencing of amplified short cDNA regions in individual reads.

The technological transition from the sequencing of amplified cDNA or non-amplified cDNA molecules to direct RNA sequencing through nanopores has already set the foundations for advanced research regarding not only transcriptomes but specific RNA classes such as mRNAs. The direct sequencing of RNA molecules that was firstly introduced by TGS, not only represents an efficient method for the study of alternative splicing, the detection of novel mRNAs [88] and the elucidation of gene expression patterns [75,78,89,90,91,92,93] but most importantly enables the accurate identification of RNA modified bases [94]. These chemical modifications of bases that occur in many classes of RNAs, including tRNAs [95], rRNA [96] and mRNAs [97], have emerged as key players in regulating pre-mRNA splicing [98], nuclear export, mRNA stability and localization as well as translation efficiency [99,100]. Although the transcriptome-wide analysis of RNA modifications through ONT’s direct RNA sequencing approach is still challenging, mainly due to the lack of specialized bioinformatics tools, it is undoubtedly the most promising method for dissolving not only the epitranscriptomic profile but also its multiple roles on cellular homeostasis and pathophysiology.

#### 6.2.2. Small RNA Sequencing

Nanopore sequencing platforms also provide approaches for the detection of small RNAs, such as miRNAs and tRNAs, allowing not only the distinct characterization of these RNA molecules but also the characterization of their expression profile in any sample of interest [101,102]. TGS has enhanced research around small RNAs by discriminating miRNA isoforms, detecting epigenetic modifications and quantifying their abundance in cells (Table 2) [102]. Direct small-RNA sequencing is, undoubtedly, the first step for studying the regulatory role of small RNAs into cells and decoding their association with human malignancies and other diseases [102].

### 6.3. Epigenetics and Metagenomics

TGS technologies have already revealed stunning advances in the research fields of epigenetics and metagenomics, being the most promising tool for the enrichment of our knowledge regarding the mechanisms that mediate chemical DNA modifications or modifications of DNA-associated proteins [103]. In the last decade, NGS was thought to be the ideal methodology for capturing chemically modified genomic regions [29]. However, the innovative TGS methodologies tend to replace the previously established approaches since they offer an unbiased real-time overview of the epigenomic alterations at a single-molecule level [17].

The key advantage of both PacBio and ONT platforms is the implementation of genome-wide sequencing analysis, and the generation of sequencing reads with greater overlaps that enhance de novo assembly. More importantly, PCR-free workflows that come with TGS technology facilitate the direct detection of epigenetic modifications and therefore reduce most of the time-consuming preparation steps. TGS platforms have introduced new advantages over methylation studies by providing genomic coverage with lower GC bias, identifying CpG islands at lower read depth [104,105] and enabling greater experimental reproducibility [106].

The novel features of TGS methodologies generate new insights into the profiling of microbial populations. TGS platforms can be used to perform rapid workflows for full-length 16S sequencing, providing real-time identification of the existing bacterial species in any sample of interest [107]. Several studies have already confirmed the capability of PacBio to provide high-throughput metagenome sequencing with highly accurate long HiFi reads. Briefly, full-length 16S rRNA sequencing is suitable for the cost-effective and real-time screening of microbial communities that simplifies the analysis and provides an in-depth phylogenetic resolution. Alternatively, each HiFi read can identify up to 8 full-length genes and provides an unbiased compositional and functional characterization of the existing microbiome, decoding with superior resolution the metagenomic profile of the sample. Finally, among PacBio’s metagenomics applications is the generation of complete genome assemblies from microbial communities involving multiplexing approaches [108].

In contrast, ONT offers metagenomics research by numerous approaches, including whole-genome and targeted sequencing, as well as metatranscriptomics and metaepigenomics analysis [109,110]. Interestingly, recent metagenomic studies have confirmed that at the genus, and particularly at the species level, ONT platforms report greater taxonomic resolution than NGS, and therefore represent a better tool for this type of analysis [111]. Rapid and real-time sequencing permits accurate discrimination of bacterial clusters as well as employs species-level functional profiling due to automated bioinformatics analysis that is provided by ONT’s software. Nanopore sequencing has brought revolutionary changes for screening bacterial populations since it allows the implementation of sequencing runs on the spot, using the portable MinION instruments. It should be mentioned that the prominent approach of 16S sequencing requires 10 ng gDNA as input quantity and supports the simultaneous sequencing of multiple samples in a single run.

Undoubtedly, the high-quality produced data by the long sequencing reads of PacBio and ONT platforms, as well as the simplified bioinformatic analysis, have paved the way for a new era in genomics and transcriptomics. Finally, both DNA-seq and RNA-seq have created a fertile ground for additional innovation in the revolutionizing space of epi- and metagenomics research.

## 7. Third-Generation Sequencing in Clinical Diagnostics

The employment of metagenomics through TGS platforms has significantly facilitated the characterization of pathogens, resulting in the accurate detection of infectious diseases [112,113]. Recently, nanopore sequencing was imported to clinical diagnostics for the rapid variant detection of SARS-CoV-2 [113]. Specifically, LamPORE constitutes an in vitro diagnostic assay for accurate and scalable detection of the SARS-CoV-2 virus [114,115]. This newly introduced test has multiplexing options for simultaneous sequencing of more than 500 RNA samples with great sensitivity and specificity (>99%). On the other hand, GenDx has incorporated PacBio Sequel II System into clinical practice for the HLA gene profiling. The approach enables long-read DNA sequencing for accurate identification of HLA allelic diversity. Moreover, both PacBio and ONT platforms have the capacity to detect large structural variants and complex genomic events, applications that enhance their significance to clinical diagnostics. The marriage of the remarkable applications of third-generation sequencing to clinical diagnostics is the key for the future of both life sciences and medicine [112].

## 8. Limitations of Third-Generation Sequencing and Future Challenges

Although TGS technologies introduced a wide spectrum of both research and clinical applications, both PacBio and ONT share critical limitations that need to be overcome. The major drawback of these technologies is the high error rate that is observed in every sequencing run. Due to the high percentage of errors that occurred during sequencing (~15%), TGS is not the recommended technology for the accurate detection of SNPs or point mutations, and as a result, NGS still remains the best technology for mutational analysis (Table 2). Improvements in the sequencing chemistry of TGS platforms are about to reduce the high error rates and, therefore, enhance the accuracy of sequencing [40]. Since TGS represents a new entry in sequencing approaches, the development of additional bioinformatics tools and algorithms necessary for downstream analysis remains a challenging task [51]. The insufficiency of pipelines for analyzing TGS datasets is a stumbling block to the efficient exploitation of the tremendous amount of data that is included in the produced HDF5 files.

## 9. Concluding Remarks

The fast-growing TGS approaches have motivated an astonishing number of technological advances and breakthroughs in the world of sequencing. Although TGS counts only a few years of existence, it has already established large-scale DNA-seq and RNA-seq applications that have elevated sequencing to a higher level [116]. The expected availability of future novel workflows, such as the forthcoming multiplexing option for the direct RNA sequencing approach, will provide further flexibility in terms of assay designing and will vigorously enrich the wide spectrum of the already existing sequencing applications that can be implemented. Overcoming the most significant limitation of the high error rates represents, without any doubt, the most serious challenge for the coming years. As a matter of fact, the newly launched nanopore flow cells, which are characterized by R10 chemistry, have already reduced the error rates, generating high-quality data that is similar to that observed from NGS platforms (accuracy > 99%). Based on the overall impact that TGS has demonstrated so far on the scientific field of genomics, potential implementation of future sequencing runs with such accuracy will certainly terminate the NGS era, eliminating the only advantage that NGS still withholds, the accurate variant calling and the efficient identification of point mutations.

Nevertheless, it should be mentioned that although TGS platforms provide increased throughput, the thorough analysis of the derived datasets remains a challenge. Although in the last few months, an increased number of bioinformatics tools and specialized algorithms have popped up, there are still limited pipelines and in silico workflows available for specific applications, including direct RNA sequencing. Undoubtedly, both the production of highly accurate reads and the introduction of further bioinformatics tools are needed, not only for the rapid evolution of TGS, but also for the future establishment of novel platforms that will correspond to an enhanced version of TGS or, even better, to a brand-new fourth-generation sequencing technology with unprecedented characteristics.

## Figures and Tables

**Table 1:** Comparison between the two TGS platforms, PacBio and ONT.

Platform	Sequencer	Average Read Length	Error Rate Per Read	Run Time	Maximum Throughput
PacBio	PacBio RS II	10–15 kb	10–15%	0.5–4 h	10 Gb
	Sequel System	10–15 kb	10–15%	≤20 h	10 Gb
	Sequel II System	10–15 kb	10–15%	≤30 h	500 Gb
	Sequel IIe System	10–15 kb	10–15%	≤30 h	500 Gb
ONT	MinION Mk1B	>4 Mb	~13%	1 min–72 h	50 Gb
	MinION Mk1C	>4 Mb	~13%	1 min–72 h	50 Gb
	GridION Mk1	>4 Mb	~13%	1 min–72 h	250 Gb
	PromethION 24	>4 Mb	~13%	1 min–72 h	7 Tb
	PromethION 48	>4 Mb	~13%	1 min–72 h	14 Tb

**Table 2:** Presentation of the major applications of TGS as well as the most suitable sequencing platform for each research interest.

Method	Application	Research Interest	Optimal Technology
**DNA** **sequencing**	Whole-genome sequencing	De novo genome assembly	PacBio/ONT
		Mutational analysis	NGS
		Extended structural variations	PacBio/ONT
		Haplotyping	PacBio/ONT
		DNA modifications	PacBio/ONT
	Whole-exome sequencing	Detection of small indels	NGS
		Detection of SNPs	NGS
		Detection of CNVs	PacBio/ONT
		Mutational analysis	NGS
	Targeted sequencing	Construction of gene panel for SNPs	NGS
		Construction of gene panel for small indels	NGS
		Construction of gene panel for extended structural variations	PacBio/ONT
		Identification of novel mRNA isoforms	PacBio/ONT
		Detection of infectious diseases	PacBio/ONT
	Whole-transcriptome sequencing	Identification of full-length transcripts	PacBio/ONT
		Characterization of fusion transcripts	PacBio/ONT
**Direct RNA sequencing**	Direct mRNA sequencing	Detection of RNA modifications	ONT
		Detection of full-length transcripts	ONT
		Characterization of fusion transcripts	ONT
		Characterization of RNA viruses	ONT
	Direct miRNA sequencing	Identification of novel miRNAs	ONT
		Detection of specific RNA modifications	ONT

**Table 3:** Presentation of the available PacBio’s and ONT’s sequencing approaches accompanied by their cost and additional information for the library preparation workflows. Of note, for the implementation of a sequencing run, the price of PacBio’s SMRT cell and ONT’s flow cell should be included in the overall cost.

Platform	Approach	Average Cost	Preparation Time	Input Amount	PCR Required
PacBio	SMRTbell Express for large DNA inserts	~$730	4 h	1–5 μg	No
	SMRTbell Express for ultra-low DNA inputs	~$850	5–8 h	5–20 ng gDNA	Yes
	CRISPR-Cas 9 system	*	>10 h	5 μg gDNA	No
	Iso-seq	~$500	8 h	300 ng total RNA	Yes
	16S amplicon sequencing	~$450	PCR + 4 h	25 pg–2.5 ng gDNA	Yes
ONT	Rapid DNA sequencing	~$200	10 min	400 ng gDNA	No
	DNA ligation sequencing	~$350	60 min	1000 ng dsDNA	No
	Cas9 Sequencing Kit	*	110 min	1–10 µg dsDNA	No
	PCR-cDNA sequencing	~$430	165 min	1 ng poly-A+ RNA (or 50 ng total RNA)	Yes
	Direct cDNA sequencing	~$400	275 min	100 ng poly-A+ RNA	No
	Direct RNA sequencing	~$350	105 min	500 ng poly-A+ RNA	No
	16S sequencing	~$350	PCR + 10 min	10 ng gDNA	Yes

* The average cost depends on the target.

## Data Availability

No new data were created or analyzed in this study. Data sharing is not applicable to this article.
